# Ferric citrate for the treatment of hyperphosphatemia and anemia in patients with chronic kidney disease: a meta-analysis of randomized clinical trials

**DOI:** 10.1080/0886022X.2022.2094273

**Published:** 2022-08-01

**Authors:** Li Li, Xin Zheng, Jin Deng, Junlin Zhou, Jihong Ou, Tao Hong

**Affiliations:** a The First Affiliated Hospital, Hengyang Medical School, Department of Nephrology, University of South China, Hengyang, China, Hengyang, China; b Department of Nephrology, Zhuzhou Central Hospital, Zhuzhou, China; c The First Affiliated Hospital, Hengyang Medical School, the Health Management Center, University of South China, Hengyang, China, Hengyang, China; d The Second Affiliated Hospital, Hengyang Medical School, Department of Endocrinology and Metabolism, University of South China, China, Hengyang, China

**Keywords:** Ferric citrate, hyperphosphatemia, anemia, chronic kidney disease, meta-analysis

## Abstract

**Background:**

Hyperphosphatemia and anemia, which are common complications of chronic kidney disease (CKD), can independently contribute to cardiovascular events. Several previous studies have found that the iron-based phosphate binder, ferric citrate (FC), could be beneficial to both hyperphosphatemia and anemia.

**Methods:**

Relevant literature from PUBMED, EMBASE, the Cochrane Central Register of Controlled Trials (CCRCT) and MEDLINE databases were searched up to 21 February 2022, in order to conduct a meta-analysis to investigate the efficacy, safety and economic benefits of ferric citrate treatment in CKD patients with hyperphosphatemia and anemia. The meta-analysis was conducted independently by two reviewers using the RevMan software (version 5.3).

**Results:**

In total, this study included 16 randomized clinical trials (RCT) involving 1754 participants. The meta-analysis showed that ferric citrate could significantly reduce the serum phosphorus in CKD patients compared to the placebo control groups (MD −1.76 mg/dL, 95% CI (−2.78, −0.75); *p* = 0.0007). In contrast, the difference between ferric citrate treatment and active controls, such as non-iron-based phosphate binders, sevelamer, calcium carbonate, lanthanum carbonate and sodium ferrous citrate, was not statistically significant (MD − 0.09 mg/dL, 95% CI (−0.35, 0.17); *p* = 0.51). However, ferric citrate could effectively improve hemoglobin levels when compared to the active drug (MD 0.43 g/dL, 95% CI (0.04, 0.82); *p* = 0.03) and placebo groups (MD 0.39 g/dL, 95% CI (0.04, 0.73); *p* = 0.03). According to eight studies, ferric citrate was found to be cost-effective treatment in comparison to control drugs. Most of the adverse events (AE) following ferric citrate treatment were mild at most.

**Conclusion:**

Collectively, our review suggests that iron-based phosphate binder, ferric citrate is an effective and safe treatment option for CKD patients with hyperphosphatemia and anemia. More importantly, this alternative treatment may also less expensive. Nevertheless, more scientific studies are warranted to validate our findings.

## 1. Introduction

The global burden of chronic kidney disease (CKD) is associated with high economic costs as well as rapidly rising morbidity and mortality. CKD is projected to become one of the leading causes of reduced life expectancy worldwide within the next 20 years [1–4]. Aggravatingly, chronic kidney disease is expected to exacerbate the financial strain on healthcare. Currently in high-income countries, the cost of dialysis and renal transplants amounts to around 3% of the annual health budget [5,6].

There appears to be an association between elevated serum phosphorus levels and increased mortality in CKD patients. Moreover, hyperphosphatemia has been linked to an increased risk of cardiovascular disease, the promotion of secondary hyperparathyroidism, soft-tissue calcification, decreased bone density, and a faster decline in kidney function [7–11]. In addition to dietary counsel, phosphate binders are essential in the management of hyperphosphatemia. Although all commonly used phosphate binders are effective in reducing serum phosphorus levels, they differ in tolerability, potential side effects [12–14]. For instance, calcium-containing phosphate binders are more likely to cause hypercalcemia, while non-calcium-based phosphate binders, such as sevelamer, are more expensive.

Anemia is another inevitable complication of CKD. Currently, the treatment of anemia in CKD is centered around using erythropoiesis-stimulating agents (ESAs) and intravenous iron injections [15]. An increased iron supplement is required because of ESAs use and ongoing blood loss, which exacerbate iron depletion, exceeding the amount available from the bone marrow. Unfortunately, several traditional oral iron formulations, such as iron gluconate, are ineffective as iron supplemental, which intravenous iron is associated with a relatively high incidence of adverse effects [16].

A previous study indicated that these CKD complications might be associated with each other, given that iron-deficiency anemia leads to upregulation of fibroblast growth factor-23 (FGF23), a hormone whose primary function is to regulate serum phosphate levels [17,18]. However, none of the commonly used phosphate binders have been shown to be effective in increasing hemoglobin levels. Notably, recent studies have found that ferric citrate not only reduces serum phosphorus levels by preventing phosphate absorption from the gastrointestinal tract, but it could also provide iron to ameliorate anemia [19]. Ferric citrate is an oral, calcium-free, iron-based phosphate binder that was approved by the USA Food and Drug Administration on 5 September, 2014. In fact, ferric compounds were shown to bind to dietary phosphate and reduce phosphorus absorption when used to treat anemia in the early 1940s [20–22]. Therefore, we conducted a meta-analysis to evaluate the efficacy and safety of ferric citrate in treating in CKD-related complications.

## 2. Materials and methods

### 2.1. Inclusion criteria and exclusion criteria

The inclusion criteria of our review were as follows: (1) population: CKD patients with hyperphosphatemia, including dialysis and non-dialysis patients; (2) intervention: treatment with iron citrate; (3) comparators: treatment with control drugs, including placebo and positive drugs; (4) at least one of the following outcome indicators was reported in the final patient evaluation: serum phosphorus, hemoglobin, serum iron, calcium, transferrin saturation and adverse events; (5) all articles included in this paper were randomized controlled trials. If a study was a review, a commentary article, a supplement, an editorial, or a conference article, it was excluded.

### 2.2. Search strategy

We searched all records in PUBMED, EMBASE and the Cochrane Central Register of Controlled Trials (CCRCT) databases up to 21 February 2022, to retrieve RCTs investigating ferric citrate treatment in CKD patients. The following search terms were applied in different databases: ferric citrate, chronic kidney disease and related subject terms. The search strategies for each database are listed in “Supplement material 1 Search strategy used in each database searched”.

### 2.3. Study selection and data extraction

Two independent reviewers (Li Li and Xin Zheng) conducted the study selection and data extraction. The initial assessment of the studies was based on title and abstract screening. If either reviewer identified a study as potentially relevant, the full text of the article was obtained for further consideration. All included studies were strictly selected according to the inclusion criteria. The following were obtained from each included study: (1) study characteristics (first author, publication year, group, participant number, treatment period, and country); (2) study participant characteristics (age, gender ratio, and baseline parameters); (3) study outcomes (efficacy, safety and economic outcomes).The last measurement value if multiple measurements were provided during the follow-up period, and the median dose was recorded if different dosages were tested in the same study. When there was disagreement, a consensus was reached through discussion.

### 2.4. Quality assessment methods

The Cochrane bias risk assessment tool provided by the Cochrane Collaboration was applied to assess the quality of each included RCT. There are six items on the assessment tool: (1) whether the randomization method is correct; (2) whether the allocation is hidden; (3) blinding method; (4) data bias; (5) selective reporting bias; (6) other biases.

### 2.5. Statistical analysis

We performed all analyses using the RevMan software (version 5.3, http://ims.cochrane.org/RevMan/download). Continuous variables in the same unit of measurement were expressed as the mean difference (MD) with 95% confidence intervals (CIs). Risk ratios (RRs) and 95% CIs were calculated for dichotomous variables. A p-value < 0.05 was considered statistically significant. The heterogeneity of the included studies was analyzed using the Chi-square test and the I^2^ statistic. A p-value < 0.1 or I^2^>50% indicated significant heterogeneity. If significant heterogeneity was observed, we used a random-effect model for data analysis. Otherwise, a fixed-effect model was adopted.

## 3. Results

### 3.1. Search results

**Figure 1.**
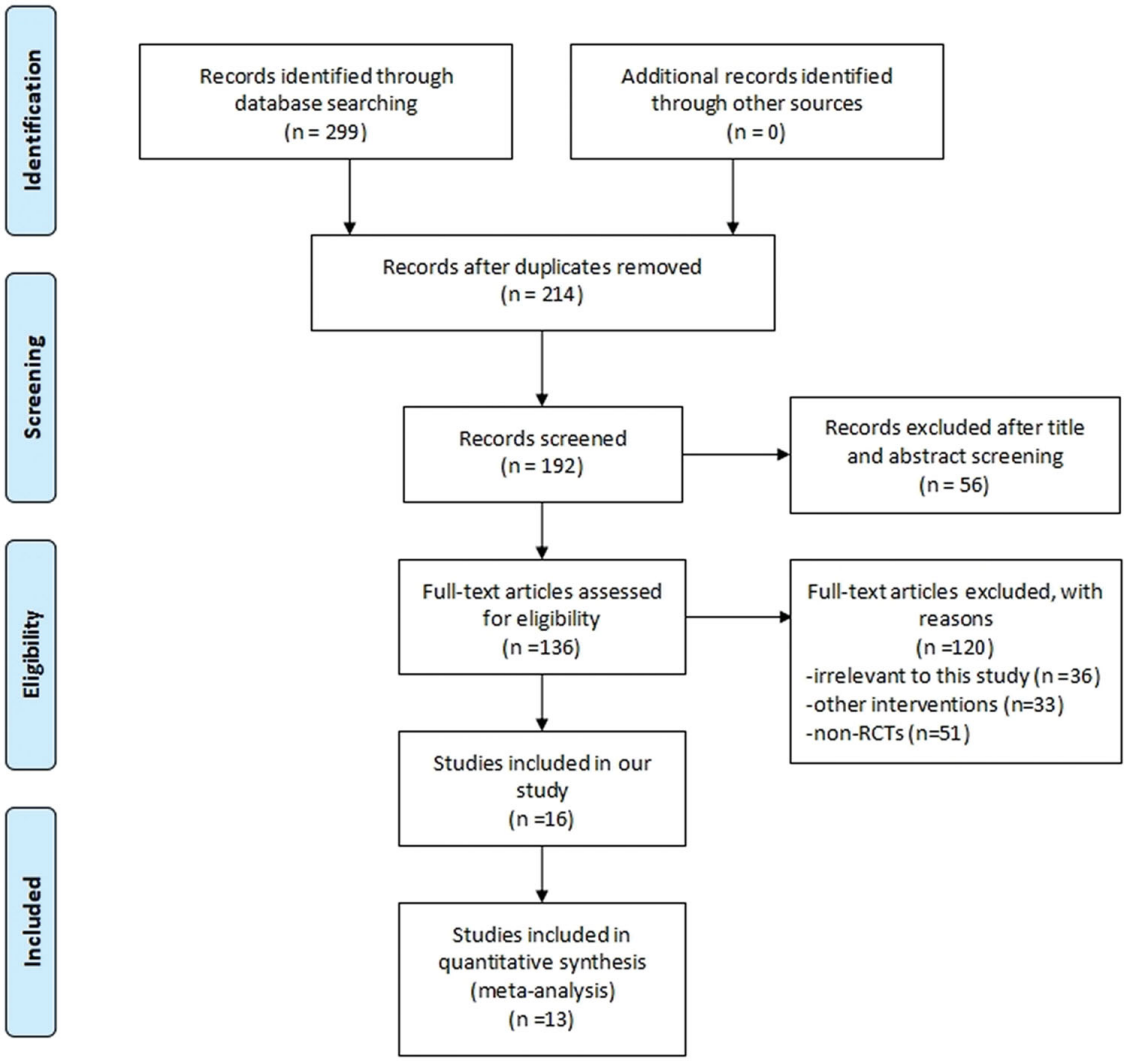
Selection process for study inclusion.

A total of 299 articles were screened using our search strategy. Following strict application of the inclusion criteria, 16 studies satisfied our inclusion criteria and were eventually included in our study. Importantly, 7 studies [23–29] included in the final analyses were based on three different trials, but each study focused on different stages of the trials and described different observation parameters. A total of 13 studies were ultimately included in our meta-analysis. The study selection process is depicted in Figure 1.

### 3.2. Risk of bias assessments

**Figure 2.**
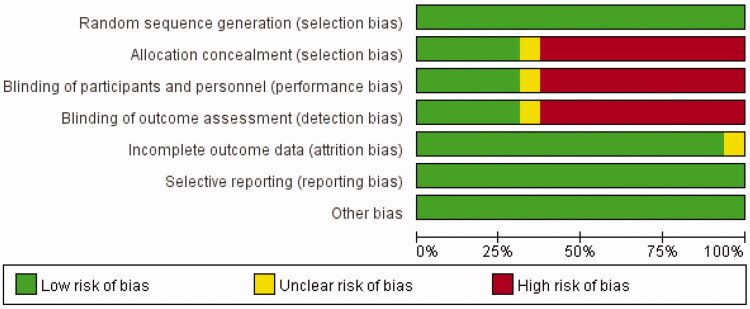
The results of the study quality assessment.

The majority of the 13 included studies were randomized controlled trials (RCTs) that used appropriate randomization methods and provided concrete descriptions of withdrawals and dropouts. Only 5 studies used a double-blinded design, and the specific methods were not described. The Risk of bias findings are listed in Figure 2.

### 3.3. Baseline characteristics of the included studies

The 13 included RCTs had a total of 1754 participants from various countries and ethnicities. The demographic characteristics were comparable among groups, and the observation parameters at baseline were balanced. The basic characteristics of the included studies at baseline are listed in Table 1.

**Table 1.** Baseline characteristics of the included studies.

Study	Groups	Numbers (n)	Age (years)	Gender (M/F)	Country	CKD stage	Treatment period(weeks)
Block. 2015	FC (5.1 g/day)	*n* = 75	66 ± 12	22/50	Not mentioned	CKD stage 3-5（include both non-dialysis and dialysis patients）	12
	PC (5.2 g/day)	*n* = 74	64 ± 14	26/43			
Block.2019	FC (Not mentioned)	*n* = 117	65.6 ± 11.2	41/76	Not mentioned	NDD-CKD	16
	PC (Not mentioned)	*n* = 115	65.3 ± 13.0	44/71			
Fishbane.2017	FC (5.0 g/day)	*n* = 117	65.6 ± 11.2	76/41	America	NDD-CKD	24
	PC (5.1 g/day)	*n* = 116	65.2 ± 13.1	71/45			
Iguchi.2018	FC (750 mg/day)	*n* = 17	74.76 ± 9.02	6/11	Japan	CKD stage 3-5（include both non-dialysis and dialysis patients）	12
	AC (Sodium ferrous citrate, 50 mg/day)	*n* = 14	66.71 ± 9.31	7/7			
	PC (Not mentioned)	*n* = 9	66.44 ± 0.92	2/7			
Lewis.2015	FC (1 g)	*n* = 292	56.0(45.0 ∼ 63.0)	183/109	America	CKD stage 5 (dialysis patients)	52 4
	AC (Calcium acetate, 667 mg or Sevelamer carbonate, 800 mg)	*n* = 149	54.0(45.0 ∼ 63.0)	87/62			
	FC (Not mentioned)	*n* = 96	54.0(45.0 ∼ 62.5)	54/142			
	PC (Not mentioned)	*n* = 96	56.0(48.5 ∼ 62.0)	56/40			
Lee. 2014	FC (4 g/day)	*n* = 75	53.4 ± 11.7	47/28	China	ESRD (dialysis patients)	8
	FC (6 g/day)	*n* = 72	56.4 ± 10.5	41/31	Taiwan		
	PC (Not mentioned)	*n* = 36	53.0 ± 11.8	25/11			
Maruyama.2018	FC (1,500(750–1,500) mg/day)	*n* = 30	62.7 ± 13	21/9	Japan	CKD stage 5(dialysis patients)	24
	AC (Lanthanum carbonate, 1,500 (750–1,812) mg/day)	*n* = 30	63.6 ± 11.8	20/10			
Rodby.2014	FC (1 g)	*n* = 292	54.9 ± 13.4	183/109	The United States and Israel	CKD requiring hemodialysis	52
	AC (calcium acetate, 667 mg or sevelamer, 800 mg)	*n* = 149	53.7 ± 13.0	87/62			
Rodby.2015	FC (1 g)	*n* = 292	54.9 ± 13.4	183/109	The United States and Israel	CKD requiring hemodialysis	52
	AC (calcium acetate, 667 mg or sevelamer, 800 mg)	*n* = 149	53.7 ± 13.0	87/62			
Umanath.2015	FC (1 g)	*n* = 292	54.9 ± 13.4	183/109	The United States and Israel	CKD requiring hemodialysis	52
	AC (calcium acetate, 667 mg or sevelamer, 800 mg)	*n* = 149	53.7 ± 13.0	87/62			
Van Buren.2015	FC (1 g)	*n* = 292	54.9 ± 13.4	183/109	The United States and Israel	CKD requiring hemodialysis	52
	AC (calcium acetate 667 mg or sevelamer, 800 mg)	*n* = 149	53.7 ± 13.0	87/62			
Yang.2002	FC (3 g/day)	*n* = 45	52.5 ± 11.8	22/23	China	CKD requiring hemodialysis	8
	AC (Calcium carbonate, 3 g/day)				Taiwan		
Yokoyama. 2012	FC (1.5 g/day)	*n* = 49	60.9 ± 8.9	30/19	Japan	CKD requiring hemodialysis	4
	FC (3 g/day)	*n* = 50	58.6 ± 12.3	32/18			
	FC (6 g/day)	*n* = 45	58.1 ± 10.6	31/14			
	PC (Not mentioned)	*n* = 48	62.7 ± 11.0	27/21			
Yokoyama.2013	FC (1.5-6.0 g/day)	*n* = 116	60.2 ± 10.7	73/42	Japan	NDD-CKD	12
	AC (Sevelamer, 3.0-9.0 g/day)	*n* = 114	61.4 ± 9.5	72/38			
Yokoyama. 2014	FC (1.5 g/day)	*n* = 60	65.3 ± 10.2	33/24	Japan	NDD-CKD	12
	PC (Not mentioned)	*n* = 30	64.5 ± 13.5	17/12			
Yokoyama.2019	FC (1500 mg/ day)	*n* = 46	63.3 ± 10	30/16	Japan	CKD stage 5(dialysis patients)	24
	AC (Not mentioned)	*n* = 45	62.7 ± 12.7	36/9			

Study	Groups	Baseline serum phosphorus (mg/dL)	Baseline hemoglobin(g/dL)	Erythropoietin discontinued	Erythropoietin dose (IU/week)	Other phosphate binders discontinued
Block. 2015	FC (5.1 g/day)	4.5 ± 0.6	10.5 ± 0.8)	Yes	–	Yes
	PC (5.2 g/day)	4.7 ± 0.6	10.6 ± 1.1	Yes	–	Yes
Block.2019	FC (Not mentioned)	4.5 ± 0.9	11.3 ± 1.6	No	Not mentioned	No
	PC (Not mentioned)	4.4 ± 0.7	11 ± 1.6	No	Not mentioned	No
Fishbane.2017	FC (5.0 g/day)	4.23 ± 0.91	10.44 ± 0.73	Not mentioned	Not mentioned	No
	PC (5.1 g/day)	4.12 ± 0.68	10.38 ± 0.78	Not mentioned	Not mentioned	No
Iguchi.2018	FC (750 mg/day)	3.59 ± 0.75	11.27 ± 1.36	Not mentioned	Not mentioned	Yes
	AC (Sodium ferrous citrate, 50 mg/day)	3.56 ± 0.55	11.99 ± 1.90	Not mentioned	Not mentioned	Yes
	PC (Not mentioned)	3.59 ± 0.59	12.58 ± 1.02	Not mentioned	Not mentioned	Yes
Lewis.2015	FC (1 g)	7.20 (6.30–8.30)	11.4 (10.7–12.2)	No	Not mentioned	No
	AC (Calcium acetate, 667 mg or Sevelamer carbonate, 800 mg)	7.40 (6.20–8.50)	11.7 (10.9–12.4)	No	Not mentioned	No
	FC (Not mentioned)	5.10 (4.30–6.00)	11.4 (10.5–12.3)	No	Not mentioned	Yes
	PC (Not mentioned)	5.30 (4.50–5.90)	10.9 (10.4–11.6)	No	Not mentioned	Yes
Lee. 2014	FC (4 g/day)	7.37 ± 1.26	6.97 ± 1.06	Not mentioned	Not mentioned	Yes
	FC (6 g/day)	6.96 ± 1.08	6.98 ± 1.12	Not mentioned	Not mentioned	Yes
	PC (Not mentioned)	6.95 ± 1.15	7.33 ± 1.18	Not mentioned	Not mentioned	Yes
Maruyama.2018	FC (1,500(750–1,500) mg/day)	5.7 ± 1.0	58.5 ± 16.3	No	6,000 (3,000–8,000)	No
	AC (Lanthanum carbonate, 1,500 (750–1,812) mg/day)	5.7 ± 0.9	56.8 ± 22.2	No	6,000 (3,000–8,000)	No
Rodby.2014	FC (1 g)	7.20(6.30-8.30)	Not mentioned	Not mentioned	Not mentioned	Yes
	AC (calcium acetate, 667 mg or sevelamer, 800 mg)	7.40(6.20-8.50)	Not mentioned	Not mentioned	Not mentioned	Yes
Rodby.2015	FC (1 g)	7.20(6.30-8.30)	Not mentioned	Not mentioned	Not mentioned	Yes
	AC (calcium acetate, 667 mg or sevelamer, 800 mg)	7.40(6.20-8.50)	Not mentioned	Not mentioned	Not mentioned	Yes
Umanath.2015	FC (1 g)	7.20(6.30-8.30)	Not mentioned	Not mentioned	Not mentioned	Yes
	AC (calcium acetate, 667 mg or sevelamer, 800 mg)	7.40(6.20-8.50)	Not mentioned	Not mentioned	Not mentioned	Yes
Van Buren.2015	FC (1 g)	7.20(6.30-8.30)	Not mentioned	Not mentioned	Not mentioned	Yes
	AC (calcium acetate 667 mg or sevelamer, 800 mg)	7.40(6.20-8.50)	Not mentioned	Not mentioned	Not mentioned	Yes
Yang.2002	FC (3 g/day)	6.7 ± 1.9	9.8 ± 1.3	Not mentioned	Not mentioned	Yes
	AC (Calcium carbonate, 3 g/day)	7.2 ± 1.9	10.1 ± 1.3	Not mentioned	Not mentioned	Yes
Yokoyama. 2012	FC (1.5 g/day)	7.69 ± 1.28	10.48 ± 1.08	No	Not mentioned	Yes
	FC (3 g/day)	7.84 ± 1.29	10.44 ± 0.96	No	Not mentioned	Yes
	FC (6 g/day)	7.93 ± 1.40	10.75 ± 1.11	No	Not mentioned	Yes
	PC (Not mentioned)	7.84 ± 1.20	10.56 ± 1.00	No	Not mentioned	Yes
Yokoyama.2013	FC (1.5-6.0 g/day)	2.53 ± 0.39	10.9 ± 1.0	No	4500(3000,6000)	Yes
	AC (Sevelamer, 3.0-9.0 g/day)	2.52 ± 0.44	11.8 ± 1.5	No	Not mentioned	Yes
Yokoyama. 2014	FC (1.5 g/day)	5.66 ± 0.75	10.25 ± 1.46	Not mentioned	Not mentioned	Yes
	PC (Not mentioned)	5.57 ± 0.63	10.52 ± 1.27	Not mentioned	Not mentioned	Yes
Yokoyama.2019	FC (1500 mg/ day)	5.36 ± 1.15	10.52 ± 0.70	No	5735.4 ± 4933.3	Yes
	AC (Not mentioned)	5.15 ± 1.25	10.47 ± 0.94	No	5848.1 ± 4082.8	Yes

Abbreviation: FC: ferric citrate; PC: placebo control; AC: active drug control; CKD: chronic kidney disease; NDD: not dependent on dialysis.

### 3.4. Results for the efficacy parameters

#### 3.4.1. Serum phosphorus

**Figure 3.**
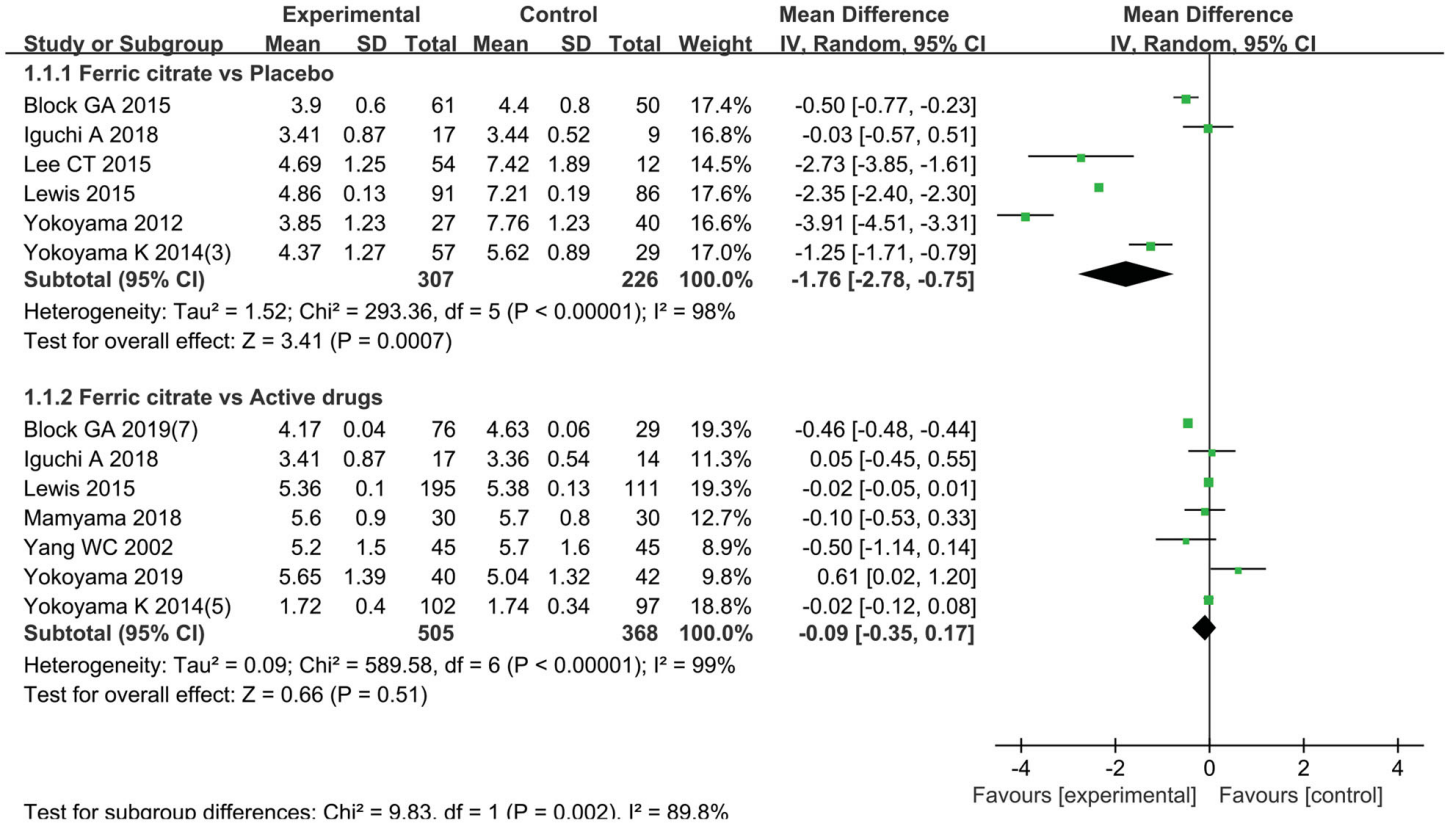
Forest plot comparing the reduction in serum phosphorous levels in patients with chronic kidney disease between the ferric citrate group and the control (including subgroup analysis of placebo and active control drug) groups.

Eleven [25,30–39] of the included studies reported the end date on serum phosphorus levels. The meta-analysis found no significant difference between the ferric citrate and active control groups (MD − 0.09 mg/dL, 95% CI (−0.35, 0.17); *p* = 0.51) [25,32–35,37,39]. On the other hand, the meta-analysis results revealed a significant decrease in serum phosphorus levels of CKD patients in the ferric citrate group compared to the placebo control group(MD − 1.76 mg/dL, 95% CI (−2.78,−0.75); *p* = 0.0007) [25,30,31,35,36,38]. The results were the mean of changes. The above finding are shown in Figure 3.

#### 3.4.2. Circulating FGF23

Serum FGF23 concentration is associated with poor outcomes in patients with advanced CKD. Five randomized controlled studies [24,32–35] suggested that ferric citrate may play a role in FGF23 reduction. For instance, in the study by Maruyama N *et al.* [34], the mean change in FGF-23 levels from baseline to the end of the study was −6,160 (−9,299 to −3,022) pg/mL in the iron citrate group and −1,118 (−4,257 to −2,021) pg/mL in the control group (*p* = 0.026). However, there is insufficient data in these articles to construct a forest plot.

#### 3.4.3. Hemoglobin

**Figure 4.**
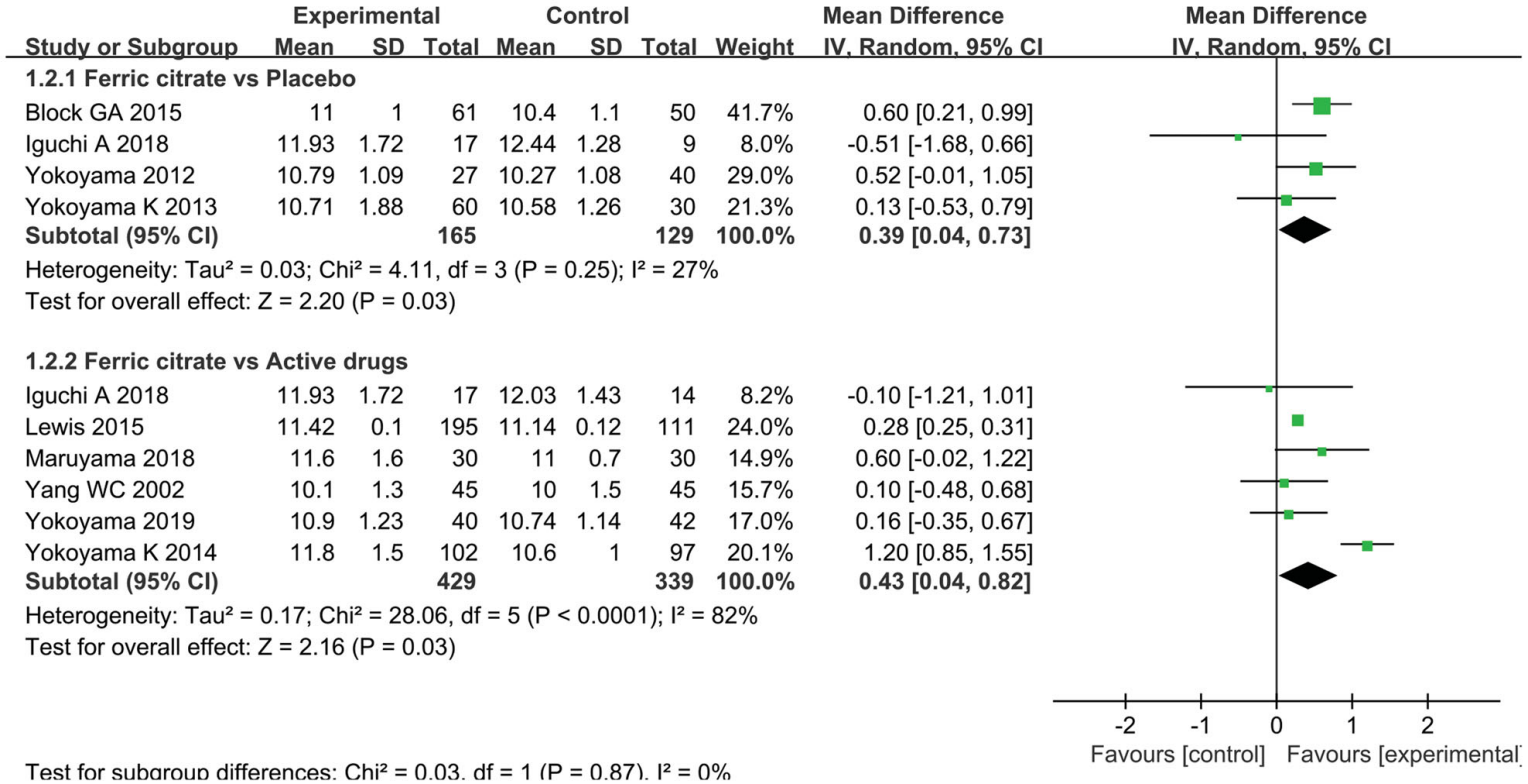
Forest plot comparing the alteration in hemoglobin levels in patients with chronic kidney disease between the ferric citrate group and the control (placebo or active control drug) group.

Only 9 of the 16 included studies discussed the end date on hemoglobin levels. Among these studies, four studies [30,31,35,36] compared ferric citrate to placebo control and six [25,33–35,37,39] compared ferric citrate to active drug control. One [35] of the articles described both comparisons. A meta-analysis of these nine studies found that ferric citrate had some advantages over active control drugs(MD 0.43 g/dL, 95% CI (0.04, 0.82); *p* = 0.03) or placebo (MD 0.39 g/dL, 95% CI (0.04, 0.73); *p* = 0.03) in increasing hemoglobin levels. The above results are demonstrated in Figure 4.

#### 3.4.4. Other related parameters

Seven studies [29,33–37,39] were conducted to evaluate whether ferric citrate resulted in hypercalcemia. The meta-analysis results revealed that there was no statistically significant difference between the ferric citrate and the placebo groups (MD 0.15 mg/dL, 95% CI (−0.1, 0.4); *p* = 0.25) [35,36] or the positive drug group (MD −0.11 mg/dL, 95% CI (−0.28, 0.05); *p* = 0.19) [29,33–35,37,39]. To more comprehensively evaluate the efficacy of ferric citrate in treating anemia, the parameters of serum ferritin levels (SF) [29,30,33,35–37], transferrin saturation (TSAT) [25,30,31,33–36] and serum iron (Fe) [31,35–37] were also examined in our meta-analysis. Ferric citrate significantly improved in serum ferritin levels (SF) compared to the placebo control group (MD 79.78 ng/mL, 95% CI (14.71, 144.86); *p* = 0.02) [30,35,36] and active control group (MD 76.69 ng/mL, 95% CI (35.73, 117.64); *p* = 0.0002) [29,33,35,37]. The effects of ferric citrate on transferring saturation (TSAT) were greater when compared to the placebo control group (MD 9.97%, 95% CI (1.58, 18.37); *p* = 0.02) [30,31,35,36] and active drug control group (MD 7.15%, 95% CI (2.02, 12.28); *p* = 0.006) [25,33,34]. In contrast, serum iron (Fe) concentrations post ferric citrate treatment did not differ significantly versus no active treatment (MD −0.22 µg/dL, 95% CI (−10.42, 9.99); *p* = 0.97) [35,37] or placebo treatment (MD 15.27 µg/dL, 95% CI (−10.81, 41.36); *p* = 0.25) [31,35,36]. The meta-analysis results are shown in Tables 2 and 3.

**Table 2.** Meta-analysis findings for Ca, SF, TSAT and Fe noted in the ferric citrate group versus the placebo control group.

Parameters	Study	MD	Heterogeneity evaluation (P, I^2^)	95% CI	P value	Model
**Ca**	2	0.15	(0.01, 41%)	(−0.10, 0.40)	0.25	RE
**SF**	2	76.69	(0.009, 79%)	(35.73, 117.64)	0.0002	RE
**TSAT**	4	9.97	(<0.0001, 88%)	(1.58, 18.37)	0.02	RE
**Fe**	3	15.27	(0.0006, 86%)	(−10.81, 41.36)	0.25	RE

Abbreviation: MD: mean difference; 95% CI: 95% confidence interval; SF: serum ferritin; TSAT: transferrin saturation; Fe: iron; FE: fixed effect; RE: random effects.

**Table 3.** Meta-analysis results for Ca, SF, TSAT and Fe noted in the ferric citrate group compared to the active drug control group.

Parameters	Study	MD	Heterogeneity evaluation (P, I^2^)	95% CI	P value	Model
**Ca**	6	−0.11	(0.0006, 77%)	(−0.28, 0.05)	0.19	RE
**SF**	4	79.78	(<0.00001, 89%)	(14.71, 144.86)	0.02	RE
**TSAT**	3	7.15	(0.002, 84%)	(2.02, 12.28)	0.006	RE
**Fe**	2	−0.22	(0.41, 0%)	(−10.42, 9.99)	0.97	FE

Abbreviation: MD: mean difference; 95% CI: 95% confidence interval; SF: serum ferritin; TSAT: transferrin saturation; Fe: iron; FE: fixed-effect; RE: random-effect.

#### 3.4.5. Adverse events

The most common adverse events during treatment were gastrointestinal reactions, including diarrhea, constipation, discolored feces, abdominal distension, abdominal pain and vomiting or nausea. In addition, several cardiovascular system, respiratory system and immune system adverse events were also reported, but these were rare. Seven studies [24,29,31,33,36,38,39] that reported adverse events over the course of treatment were included in our meta-analysis. RRs and 95% CIs were calculated to evaluate the risk of adverse events. Our analysis revealed that the incidence of any adverse events（AEs）was comparable between the ferric citrate and the active drug group (RR 0.94, 95% CI 0.8 to 1.1, P 0.45). In contrast, the difference in AEs between the ferric citrate group and the placebo group was statistically significant (RR 1.19, 95% CI 1.04 to 1.37, P 0.009). There was no significant difference in diarrhea, constipation, vomiting or nausea, abdominal pain and abdominal distension occurrences between groups. However, there was a significant difference in terms of discolored feces. The reported adverse events are detailed in Tables 4 and 5.

**Table 4.** Meta-analysis results for adverse events reported in the ferric citrate group compared with the placebo control group.

AE	Study	FC	PC	Heterogeneity evaluation (P, I^2^)	RR	95% CI	P value	Model
Any AEs	4	215/396	118/230	(0.088, 0%)	1.18	(1.03, 1.35)	0.02	FE
Discolored feces	2	44/189	2/152	（0.29, 12%）	11.18	(3.39, 36.88)	<0.0001	FE
Constipation	4	34/294	17/230	（0.65, 0%）	1.71	(1.00, 2.92)	0.05	FE
Diarrhea	4	60/294	26/230	(0.01, 73%)	2.10	(0.71, 6.27)	0.18	RE
Abdominal distension	3	5/177	0/114	(0.92, 0%)	2.71	(0.46, 15.87)	0.27	FE
Vomiting or nausea	3	15/222	5/194	(0.11, 55%)	1.69	(0.27, 10.74)	0.58	RE
Abdominal pain	3	12/234	3/200	(0.31, 14%)	3.21	(1.40, 9.86)	0.31	FE

Abbreviation: FC: ferric citrate; PC: placebo control; AE: adverse events; RR: relative risk; 95% CI: 95% confidence interval; FE: fixed-effect; RE: random-effect.

**Table 5.** Meta-analysis results for adverse events reported in the ferric citrate group compared with the active drug control group.

AE	Study	FC	AC	Heterogeneity evaluation (P, I^2^)	RR	95% CI	*P* value	Model
								
Any AEs	3	230/451	186/307	(0.17, 44%)	0.94	(0.80, 1.10)	0.45	RE

Abbreviation: FC: ferric citrate; AC: active drug control; AE: adverse events; RR: relative risk; 95% CI: 95% confidence interval; RE: random effects.

#### 3.4.6. Costs

Eight studies estimated medical expenses associated with hospitalizations, cumulative ESA (erythropoiesis-stimulating agents) and intravenous iron. However, there were insufficient randomized controlled studies to construct a forest plots. Notably, the study by Yokoyama K *et al.* [31] reported that ferric citrate could potentially amount to savings of 90.51–181.01 dollars/patient/month for the treatment of hyperphosphatemia. Moreover, according to the seven other RCTs, ferric citrate has shown to be a promising drug with potential cost savings because patients receiving ferric citrate experienced fewer hospitalizations [26,32] and reduced intravenous iron and ESA usage [25,27,28,33,34] compared to patients receiving an active control drug. Together, these data suggest that ferric citrate may be economically beneficial as a treatment in CKD patients.

## 4. Discussion

The development of anemia and hyperphosphatemia in CKD patients significantly increases the medical costs, symptoms, and mortality risks of the patients, which are the frequent comorbidities during CKD [40]. Therefore new strategies and drugs need to be designed to help relieve this burden. The mechanisms of enteral absorption of iron delivered by ferric citrate may include the possibility of traditional, transcellular ferritin mediated absorption and/or citrate mediated paracellular absorption [41].

Following oral administration of FC, the dissociated ferric iron binds to phosphorus in the gastrointestinal (GI) tract and precipitates as ferric phosphate, which could be removed from the intestine to minimize the absorption of phosphate in the intestine. On the other hand, the enteral absorption of iron from FC increases serum iron, ferritin and TSAT levels, thereby increasing total hemoglobin and RBC mass [42,43]. According to several studies, FC acts as both a phosphate binder and an iron supplement in CKD patients, which is consistent with confirmed in our meta-analysis findings.

According to our results, iron citrate, an iron-based phosphate binder, is a widely effective and safe treatment option for CKD patients with hyperphosphatemia and anemia. Importantly, may also offer economic benefits to these patients. Iron citrate had significant phosphorus-lowering and anemia-correcting effects when compared to placebo. Notably, the phosphorus-lowering effect was comparable to that of traditional phosphorus-lowering drugs, while its anemia treating effect was more pronounced; hence this treatment option should be preferred. In patients with chronic kidney disease, serum FGF23 concentration is associated with iron deficiency, which increases cardiomyocyte injury and leads to systemic inflammatory responses. Besides, it is also linked to the occurrence of cardiovascular events in chronic kidney disease patients [44–46]. Collectively, our study found that iron citrate may play a role in lowering serum FGF23 serum concentrations, which may improve adverse outcomes in CKD patients.

Our review was not the first meta-analysis on ferric citrate for the treatment of patients with CKD. In 2019, a meta-analysis [47] that evaluated the effect of iron-based phosphate binders in dialysis patients was published. Although 16 studies were included in that review, the economic benefits of iron citrate were not described in the meta-analysis. Our meta-analysis has added three studies [26–28], building on the foundation of the previous meta-analysis, to assess the efficacy of ferric citrate in treating CKD-associated hyperphosphatemia and anemia. Furthermore, our meta-analysis only included comparative results between ferric citrate and the control groups, and the findings were more comparable and valuable. Compared to a meta-analysis published in 2015, our meta-analysis has several advantages. Firstly, more high-quality studies focused on ferric citrate for the treatment of patients with CKD were included. Secondly, there was little heterogeneity for several outcomes because only ferric citrate was studied since articles on other iron-based phosphate binders were insufficient. Thirdly, our study included a meta-analysis of hemoglobin levels and serum iron-related parameters involving a larger sample size, indicating that our review has greater weight than the previous review. Finally, the economic outcomes of each patient were taken into account in our review, which was not addressed in the previous review. Nevertheless, our systematic review still has several shortcomings. First, the majority of the included studies used short treatment durations, as little as 4 weeks; thus, the long-term efficacy and toxicity of ferric citrate remain unknown. Second, while the baseline patient characteristics of the included RCTs were similar, there were several heterogeneities in clinical features, resulted in an unclear risk of bias in the meta-analysis to some extent. For example, heterogeneity in treatment regimens due to different dosages among studies cannot be eliminated by conducting a subgroup analysis. Third, although our study included 16 studies with 1754 participants, only half of the studies and fewer than half of the participants were included in the meta-analysis for various reasons; this greatly limited the scale of our meta-analysis. Fourth, our article addresses economic benefits that previous articles have never covered. However, it is difficult to explain the economic advantages in patients with CKD by simple description, because there are numerous complications, particularly in CKD patients with end-stage disease. Our analysis may prompt us to delve deeper into the economic benefits since this would be advantageous for CKD patients if this drug is, in fact, cost-effective. Therefore, further scientific studies are warranted to confirm our findings. Lastly, the risk of publication bias has not been evaluated by performing a funnel plot in our review because fewer than ten studies were included for a given parameter. To avoid the above-mentioned shortcomings, additional high-quality RCTs with larger samples using the same dosage, a longer follow-up duration and a network meta-analysis should be performed. If new RCTs on this topic are conducted, it is unquestionably necessary to conduct a more comprehensive, up-to-date meta-analysis systematically describing the treatment efficacy of ferric citrate in comparison to various other types of phosphate binders.

In conclusion, the iron-based drug ferric citrate appears to be broadly effective in the treatment of hyperphosphatemia and anemia among patients with CKD. Furthermore, it may also have economic benefits.

## Data Availability

The data of this study can be obtained from the corresponding author upon rational request.

## Supplementary materials

The “PRISMA 2020 checklist” is a standardized checklist that ensures that systematic reviews and meta-analyses are implemented according to this standard.
